# Suppression of TCF4 promotes a ZC3H12A-mediated self-sustaining inflammatory feedback cycle involving IL-17RA/IL-17RE epidermal signaling

**DOI:** 10.1172/jci.insight.172764

**Published:** 2024-03-12

**Authors:** Yanyun Jiang, Dennis Gruszka, Chang Zeng, William R. Swindell, Christa Gaskill, Christian Sorensen, Whitney Brown, Roopesh Singh Gangwar, Lam C. Tsoi, Joshua Webster, Sigrún Laufey Sigurðardóttir, Mrinal K. Sarkar, Ranjitha Uppala, Austin Kidder, Xianying Xing, Olesya Plazyo, Enze Xing, Allison C. Billi, Emanual Maverakis, J. Michelle Kahlenberg, Johann E. Gudjonsson, Nicole L. Ward

**Affiliations:** 1 Department of Dermatology, Ann Arbor, Michigan, USA.; 2 Department of Dermatology, Sun Yat-sen Memorial Hospital, Sun Yat-sen University, Guangzhou, China.; 3 Departments of Nutrition and Dermatology, Case Western Reserve University, Cleveland, Ohio, USA.; 4 Department of Internal Medicine, University of Texas Southwestern Medical Center, Dallas, Texas, USA.; 5 Department of Dermatology, Vanderbilt University Medical Center, Nashville, Tennessee, USA.; 6 Department of Dermatology, University of California Davis School of Medicine, Sacramento, California, USA.; 7 Division of Rheumatology, Department of Internal Medicine, University of Michigan, Ann Arbor, Michigan, USA.; 8 Vanderbilt Institute for Infection, Immunology, and Inflammation (VI4) and Vanderbilt Center for Immunobiology (VCI), Vanderbilt University Medical Center, Nashville, Tennessee, USA.

**Keywords:** Dermatology, Immunology, Cytokines, Signal transduction, Skin

## Abstract

IL-17C is an epithelial cell–derived proinflammatory cytokine whose transcriptional regulation remains unclear. Analysis of the *IL17C* promoter region identified *TCF4* as putative regulator, and siRNA knockdown of *TCF4* in human keratinocytes (KCs) increased *IL17C*. IL-17C stimulation of KCs (along with IL-17A and TNF-α stimulation) decreased *TCF4* and increased *NFKBIZ* and *ZC3H12A* expression in an IL-17RA/RE–dependent manner, thus creating a feedback loop. ZC*3H12A (*MCPIP1/Regnase-1), a transcriptional immune-response regulator, also increased following *TCF4* siRNA knockdown, and siRNA knockdown of *ZC3H12A* decreased *NFKBIZ*, *IL1B*, *IL36G*, *CCL20*, and *CXCL1,* revealing a proinflammatory role for ZC3H12A. Examination of lesional skin from the KC-Tie2 inflammatory dermatitis mouse model identified decreases in TCF4 protein concomitant with increases in IL-17C and *Zc3h12a* that reversed following the genetic elimination of *Il17c*, *Il17ra*, and *Il17re* and improvement in the skin phenotype. Conversely, interference with *Tcf4* in KC-Tie2 mouse skin increased *Il17c* and exacerbated the inflammatory skin phenotype. Together, these findings identify a role for TCF4 in the negative regulation of IL-17C, which, alone and with TNF-α and IL-17A, feed back to decrease TCF4 in an IL-17RA/RE–dependent manner. This loop is further amplified by IL-17C–TCF4 autocrine regulation of *ZC3H12A* and IL-17C regulation of *NFKBIZ* to promote self-sustaining skin inflammation.

## Abstract 2

A negative TCF4/IL-17C/ TNF-a/ IL-17A feedback loop decreases TCF4 in an IL-17RA/RE-dependent manner and is further amplified by IL-17C/TCF4 regulation of ZC3H12A and IL-17C regulation of NFKBIZ, resulting in self-sustaining skin inflammation.

## Graphical abstract

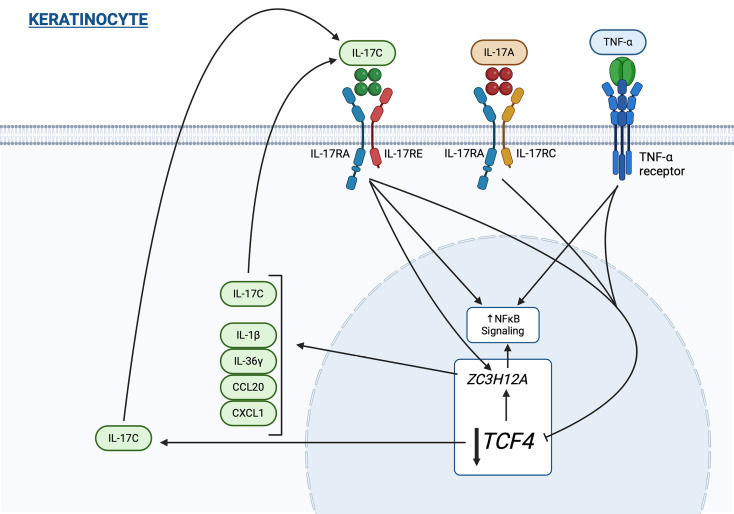

## Introduction

The skin serves as a barrier against the entry of physical agents, chemicals, and microbes (1). Keratinocytes (KCs) are the main cellular constituent of the epidermis and express multiple types of pattern-recognition receptors that are responsible for the initiation of a broad range of inflammatory responses (2). These responses are largely executed via the secretion of various proinflammatory cytokines and chemokines, which elicit feedback loops within the KC itself, as well as via skin-infiltrating immune cells that sustain inflammation and also provide targets for therapeutic strategies for a variety of inflammatory skin diseases (3). Thus, the KC is a critical cell in barrier defense as well as in the initiation and maintenance of skin inflammation.

IL-17C is a member of the IL-17 cytokine family (IL-17A-F; ref. 4). IL-17A and IL-17F are expressed by immune cells and bind to IL-17RC and IL-17RA; their transcriptional regulation and contribution to inflammatory skin diseases are well understood. IL-17C is expressed primarily by epithelial cells, including KCs, and binds to IL-17RE and IL-17RA (5, 6) to promote and amplify innate defense in epithelial cells; despite repeated implications for its role in the pathogenesis of inflammatory skin disorders, including psoriasis (Ps) and atopic dermatitis (AD) (7), its transcriptional regulation is not well understood. Notably, IL-17C is the most abundant IL-17 family cytokine in human Ps and AD skin, and lesional skin from patients with Ps has ~100-fold more IL-17C protein than IL-17A (7–9). While the role of IL-17C in skin inflammation remains unclear, overexpression of IL-17C in KCs promotes psoriasiform skin inflammation in a murine model (8), and IL-17C–deficient mice are protected from imiquimod-elicited inflammation (5). Furthermore, neutralization of IL-17C reduces skin inflammation in the IL-23–elicited mouse model of Ps and in the calcipotriol-induced (MC903) and flakey tail strain mouse models of AD, and it reduces the proinflammatory mediators HBD2, IL-36γ, and LCE3A in human lesional skin explants of Ps and atopic eczema (7, 9). IL-17C is known to be induced by stimulation of bacterial products or cytokines including TNF-α, IL-1β, IL-36γ, and IL-17C (8, 10–12), but little else is known about the regulation of IL-17C.

TCF4 is a class I bHLH transcription factor, which has been shown to play an important role in nervous system development (13) and in repression of neural inflammatory responses (14). A role for TCF4 in skin is supported by studies revealing that TCF4 contributes to epidermal development and homeostasis (15–17). During mouse development, for example, TCF4 is expressed by epidermal progenitor cells, and overexpression of *Tcf4* represses proliferation in cultured epidermal progenitors (18). Whether TCF4 contributes to skin inflammation has not been examined.

Here we report that *TCF4* negatively regulates *IL-17C* and *ZC3H12A* (also known as, MCPIP1/Regnase-1) expression in human KCs and that IL-17C further promotes the expression of *ZC3H12A* and *NFKBIZ* in an IL-17RA/RE–dependent manner. IL-17C, IL-17A, and TNF-α, alone and together decrease *TCF4*, and this further increases *ZC3H12A* and *IL17C*, strengthening the inflammatory feedback loop within the KC. This loop becomes self-sustaining as increases in *ZC3H12A* increase *NFKBIZ* and other proinflammatory cytokines, *IL1B* and *IL36G*. Using the KC-Tie2 mouse model of inflammatory dermatitis, which has increases in cutaneous IL-17C and *Zc3h12a* and decreases in epidermal TCF4, we confirm the importance of these pathways in vivo by demonstrating that genetic deletion of *Il17c* or *Il17re/ra* increases TCF4, decreases IL-17C, IL-17A and *Zc3h12a*, and eliminates the inflammatory skin phenotype. Moreover, interference with cutaneous TCF4 using topical application of *Tcf4* siRNA worsens skin inflammation by increasing cutaneous *Il17c* and *Zc3h12a* and by increasing skin infiltrating CD3^+^ T cell numbers, resulting in worsened acanthosis. These findings identify a function for TCF4 in the negative regulation of IL-17C and ZC3H12A that promotes a self-sustaining inflammatory feedback cycle within the KC, dependent on IL-17RE/RA and the autocrine production of *NFKBIZ*, *IL17C*, *IL1B*, *IL36G,* and TNF-α.

## Results

### Promoter analysis outlines IL-17C coexpressed genes and upstream motifs.

**Figure 1:**
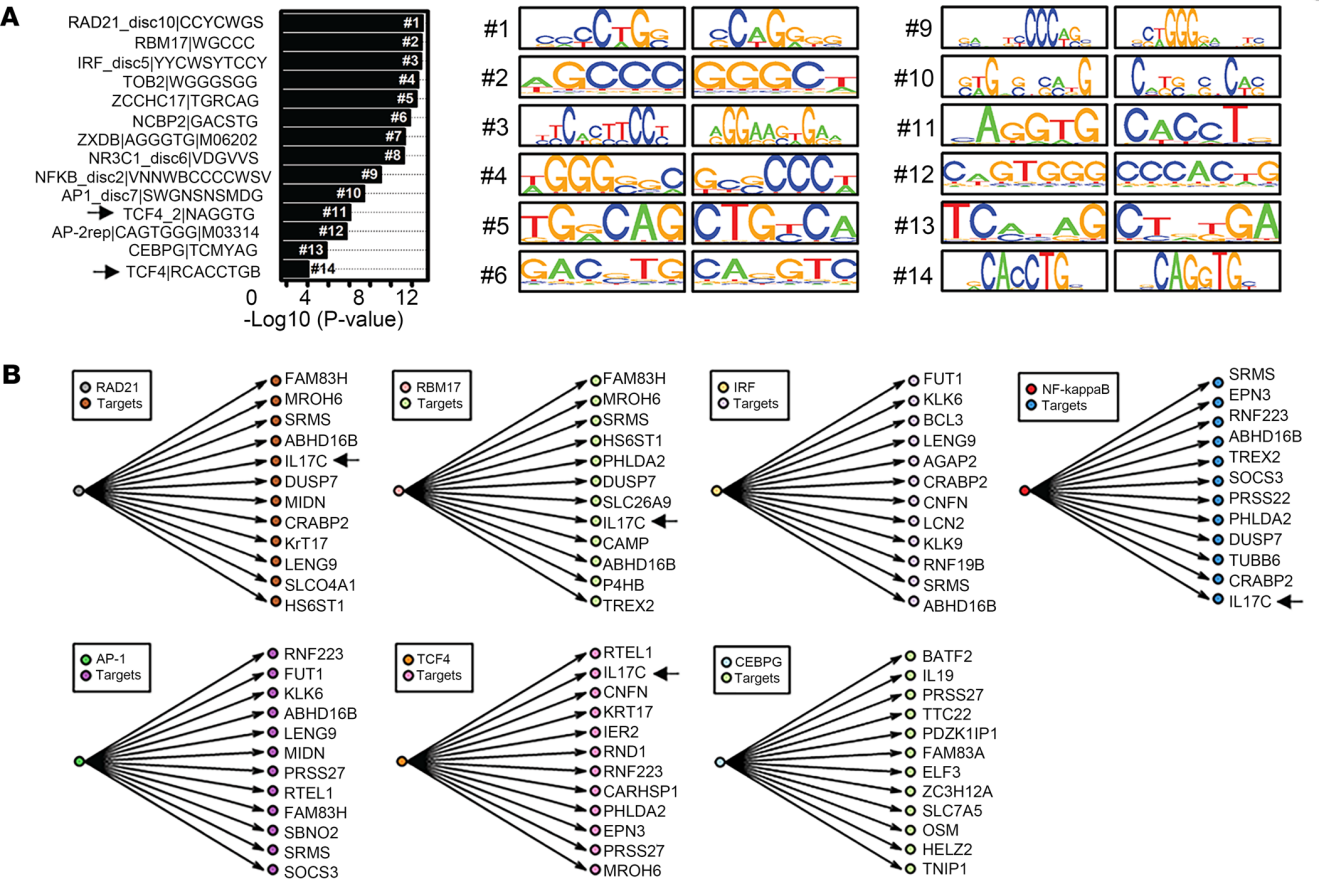
Identification of upstream motifs from the *IL17C* promoter sequence. (**A**) Motif analysis shows enriched motifs in the 10 kb region upstream of *IL17C*-correlated genes. Only motifs with at least 5 sites in the IL17C 10 kb upstream region are shown. (**B**) The top 12 predicted target genes are shown for RAD21, RBM17, IRF, NF-κB, AP-1, CEBPG, and TCF4 transcription factor motif. IL17C is a top target gene for TCF4.

To better understand the role of IL-17C in regulating skin inflammation, and to identify new pathways by which it was regulated and functioned, we analyzed the *IL17C* promoter (Supplemental Figure 1; supplemental material available online with this article; https://doi.org/10.1172/jci.insight.172764DS1). To do this, we examined *IL17C* gene expression changes using lesional skin samples from 99 data sets from patients with Ps (19) and identified 18,329 genes with detectable expression in at least 5% of samples (≥5/99, CPM > 0.25 with FPKM lower 95% VI greater than 0). Of these 18,329 genes, 6,276 genes were positively correlated with *IL17C* (*r*_s_ > 0). Genes most strongly correlated with *IL17C* expression included rho family GTPase 1 (*RND1*), nitric oxide synthase 2 (*NOS2*), E74-like ETS transcription factor 3 (*ELF3*), and ChaC glutathione-specific gamma-glutamylcyclotransferase 1 (*CHAC1*) (*r*_s_ ≥ 0.67). Other known Ps-related genes were also observed to strongly correlate, including proinflammatory cytokines *IL36A*, *IL36G*, *IL23A*, and *IL1B;* the endoribonuclease *ZC3H12A*; and transcription factors *STAT3*, *JUNB*, and *BATF2* (Supplemental Figure 1D). As a group, the 191 *IL17C*-correlated genes were most strongly enriched with respect to the Gene Ontology (GO) biological process (BP) terms “skin development”, “epidermal cell differentiation”, “immune response cell activation”, and “cornification” (Supplemental Figure 1E). With respect to 5 kb upstream regions, 4 significant DNA binding sites were identified, including RAD21 cohesin complex component (*RAD21*), RNA binding motif protein 17 (*RBM17*), and IFN regulatory factor (*IRF)* (Figure 1A). Of particular interest was the identification of DNA binding sites known to be involved in IL-17A signaling, including AP-1, NF-κB, CEBPG, and more so the discovery of 2 TCF4 binding sites highly ranked within this region (Figure 1A). Predication analyses of the top 12 targets genes of RAD21, RBM17, IRF, NF-κB, CEBPG, AP-1, and TCF4 identified IL17C as a top target for TCF4; less so for NF-κB, RAD21, and RBM17; and not at all for CEBPG, AP1, or IRF (Figure 1B).

### TCF4 negatively regulates IL-17C activity in KCs.

**Figure 2:**
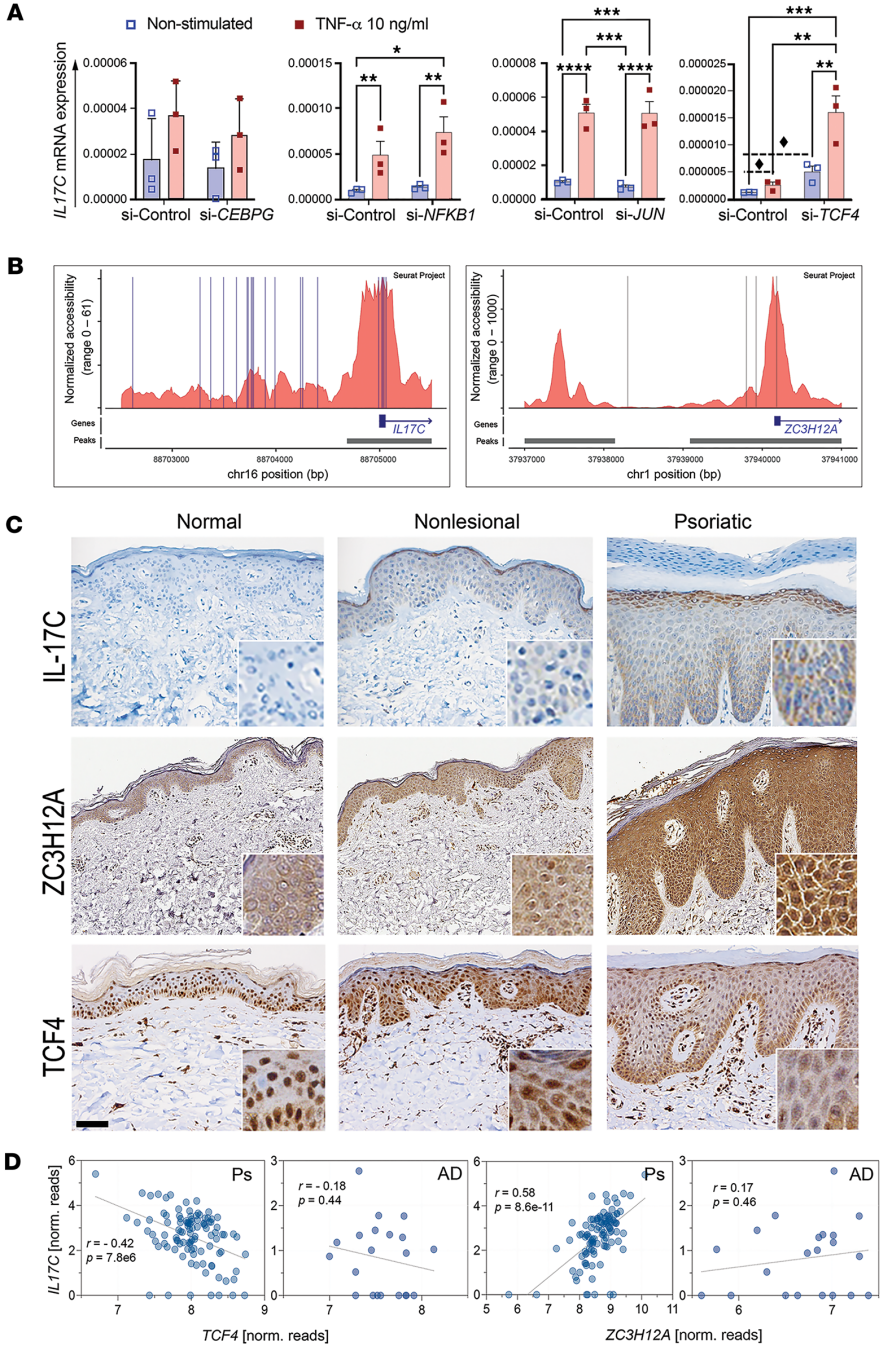
TCF4 negatively regulates IL-17C in keratinocytes. (**A**) Silencing *TCF4 (*siRNA-mediated*)* in N/TERT keratinocytes, but not *NFKB1*, *JUN*, or *CEBPG*, increases *IL17C* mRNA expression, which is further increased in the presence of TNF-α stimulation (10 ng/mL; *n* = 3, mean ± SEM, 2-way ANOVA with post hoc Tukey test. *P < 0.01; **P < 0.005, ***P < 0.001, ****P < 0.0001; dashed line and the diamond demarcate *P* < 0.05 between 2 indicated groups via Student’s *t* test (Supplemental Figure 3). (**B**) ATAC-Seq of human KCs isolated from fresh tissue biopsies identifies *TCF4* binding sites in open chromatin regions of *IL17C* and *ZC3H12A* promoters. (**C**) Representative images of healthy normal and of lesional and nonlesional Ps skin demonstrates decreases in TCF4 (nuclear localization) and increases in IL-17C and ZC3H12A staining (using IHC; stained protein appears brown in color). Insets represent higher-magnification image. Scale bar: 100 μm; 20 μm (insets). (**D**) *IL17C* expression negatively correlates with *TCF4* and positively correlates with *ZC3H12A* in lesional Ps and AD skin.

The transcriptional control of IL-17C remains poorly understood; thus, to identify regulators of IL-17C expression, we selected several of the candidates from the promoter analysis, including NFKB1, JUN, CEBPG, known transcription factors for IL-17A-IL-17RA/RC signaling, and TCF4, a novel candidate not previously studied, for functional testing. Knockdown in human KCs was completed using siRNA targeting each transcription factor, followed by stimulation with or without TNF-α. siRNA of each transcript led to significant decreases in each target gene (Supplemental Figure 2A). *IL17C* mRNA expression increased in direct response to small interfering *TCF4* (si-*TCF4*) in nonstimulated and TNF-α–stimulated conditions (Figure 2A and Supplemental Figure 3), reflecting the ability of TCF4 to negatively regulate IL-17C. No changes in *IL17C* expression were observed following the silencing of the other transcriptional regulators (Figure 2A), although we observed significant decreases in *TCF4* and increases in *NFKB1* expression in si-control KCs stimulated with TNF-α (Supplemental Figure 2A). These findings demonstrate that TNF-α decreases *TCF4* expression in KCs, and that decreases in *TCF4* increase IL-17C.

Consistent with these findings, TCF4 binding sites corresponded to open chromatin areas around the *IL17C* promoter as shown by single-cell ATAC-Seq of KCs (Figure 2B). Indeed, a motif known to interact with TCF4 (5’-CAGGTG/CACCTG-3’) was more abundant in the 2 kb promoter region of the 191 *IL17C*-correlated genes as compared with all other genes expression in lesional skin of patients with psoriasis (PP) (*P* = 5.40 × 10^–9^, FDR = 1.47 × 10^–6^) (Supplemental Figure 4). TCF4 binding sites were also identified in open chromatic areas around the *ZC3H12A* promoter. To address the potential interaction between TCF4 and IL-17C in skin, we IHC stained normal healthy skin, nonlesional Ps skin, and lesional Ps skin using antibodies directed toward TCF4, IL-17C, and ZC3H12A (Figure 2C) or control rabbit isotype (Supplemental Figure 2B). IL-17C and ZC3H12A were each abundantly expressed in Ps lesions compared with healthy skin, confirming prior reports (8, 20) (Figure 2C). In contrast, TCF4 staining showed stronger staining and nuclear localization in healthy skin and decreased staining, particularly in KC nuclei, in Ps skin (Figure 2C). Similarly, using RNA-Seq data from lesional skin samples from patients with Ps and AD (21–23), we determined that *TCF4* expression negatively correlates with *IL17C* and *ZC3H12A* and that *IL17C* positively correlates with *ZC3H12A* in a disease-specific manner (Figure 2D). Together, these data identify a role for TCF4 as a negative regulator of IL-17C and *ZC3H12A* expression in inflammatory states.

### Inflammatory cytokines negatively regulate TCF4 expression in KCs.

**Figure 3:**
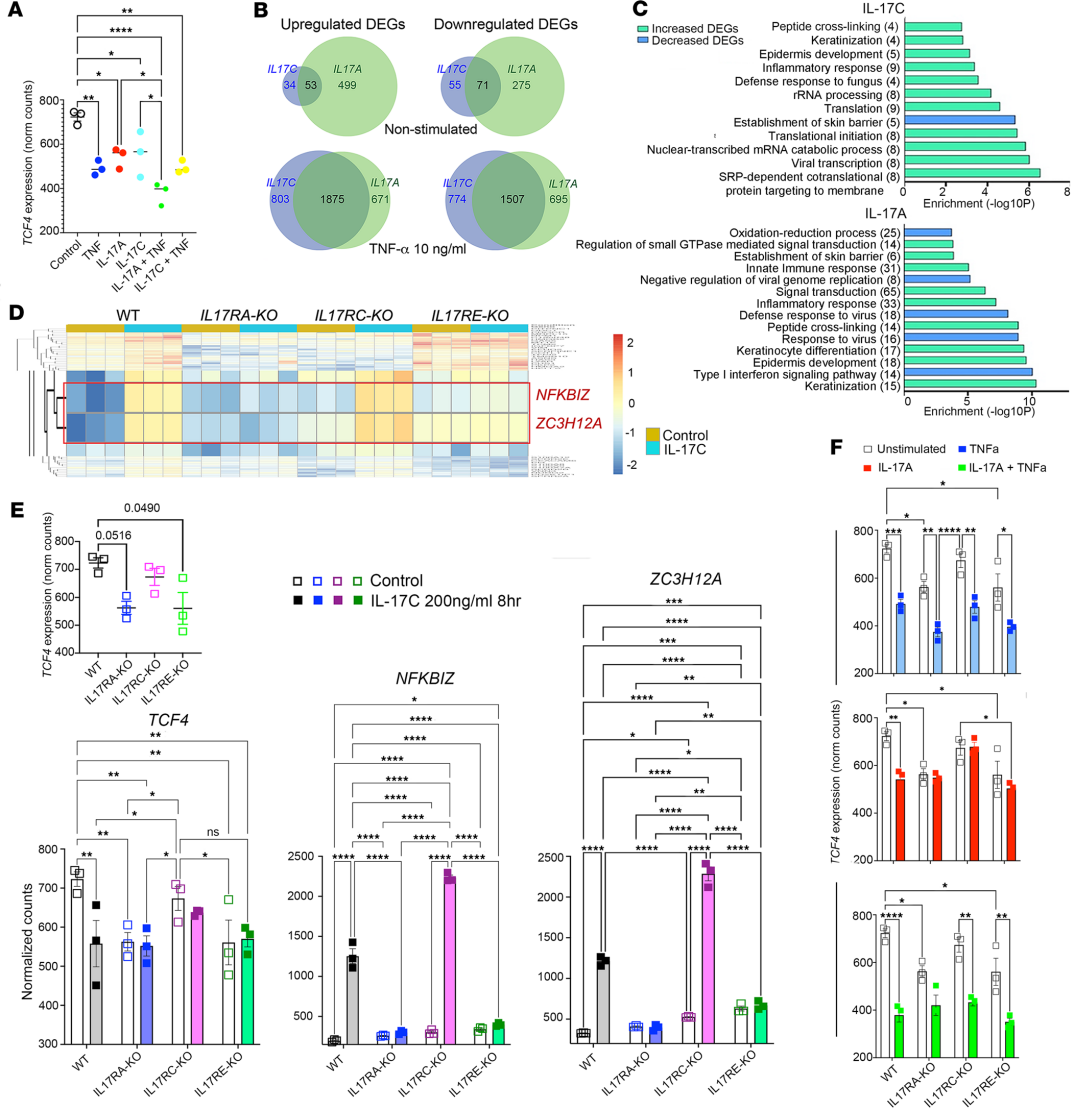
The biological role of IL-17C–IL-17RE/RA in keratinocytes. (**A**) *TCF4* decreases in KCs stimulated with IL-17C (200 ng/mL), IL-17A (20 ng/mL), TNF-α (10 ng/mL; 8 hours), and combinations of these cytokines as indicated. (**B**) Venn diagrams show the number of DEGs altered by IL-17C and IL-17A in keratinocytes with or without TNF-α stimulation. (**C**) GO BP terms. The chart shows functional categories enriched in keratinocytes induced by IL-17C and IL-17A. (**D**) Partial heatmap showing *NFKBIZ* and *ZC3H12A* increase in an IL-17C–IL17RA/RE–dependent manner. Full heatmap is presented in Supplemental Figure 8. (**E**) Top, *TCF4* decreases in KCs engineered to have no *IL17RE* and *IL17RA*. Bottom, since ZC3H12A and NFKB had also been identified in the IL-17C promotor analyses (Figure 1), we focused on these target genes. Normalized counts of *TCF4*, *NFKBIZ,* and *ZC3H12A*, following IL-17C stimulation in WT and *IL17RA-*, *IL17RC-*, and *IL17RE*-KO keratinocytes. (**F**) *TCF4* decreases in KCs stimulated with TNF-α, IL-17A, and IL-17A + TNF-α and are dependent on IL-17RE/RA expression. *n* = 3, mean ± SEM, 1-way ANOVA (**A** and **E** inset) and 2-way ANOVA (**E** and **F**) with post hoc Tukey test. *P <0.05, **P < 0.002, ***P < 0.0005, ****P < 0.0001.

The observation that TNF-α stimulation of KCs decreases *TCF4* expression and that interfering with TCF4 expression increases IL-17C (Figure 2, Supplemental Figure 2A, and Supplemental Figure 3) suggests a possible feedback cycle between inflammatory cytokines and TCF4. We sought to determine whether IL-17C could negatively regulate TCF4 gene expression, akin to that of TNF-α. Therefore, KCs were stimulated with recombinant TNF-α, IL-17A, and IL-17C and combinations of IL-17A/C and TNF-α and *TCF4* expression levels examined (Figure 3A). We confirmed that TNF-α stimulation significantly decreased *TCF4* and determined that both IL-17C and IL-17A also decreased *TCF4* gene expression, which further decreased when IL-17A was combined with TNF-α.

To identify additional mechanisms of TCF regulation, we performed similar analyses (Supplemental Figure 5) as that used to study IL-17C (Supplemental Figure 1). TCF4-correlated gene promoters showed enrichment for 5’-AAAT-3’ elements, which are recognized by several different proteins. The TCF4-correlated genes themselves show functional enrichment with respect to various developmental processes. From these analyses, the best candidate TCF4 regulatory factors proposed included paired related homeobox 1 (PRRX1, 5’-TAATT-3’), transcription factor 12 (TCF12, 5’-TGTTTRCW-3’), forkhead box O1 (FOXO1, 5’-WTGTTTAC-3’), and nuclear factor I B (NFIB, 5’-STTYGC-3’). The genes encoding these factors are highly correlated with TCF4 expression in PP samples, and the promoters of TCF4-correlated genes are enriched for elements recognized by these factors. However, unlike TCF4, *NFIB* failed to change in response to cytokine stimulation, and only modest increases in *TCF12* were observed following IL-17C + TNF-α stimulation. *FOX01* significantly decreased following stimulation with IL-17A + TNF-α and IL-17C + TNF-α, and *PRX1* decreased following stimulation with TNF-α and IL-17C + TNF-α (Supplemental Figure 6), mirroring observations with *TCF4* (Figure 3A). These changes support what others have shown, such that decreases in FOXO1 lead to increases in IL-1β (24) and *PRX1* inhibits apoptosis (25). Additional analyses of TCF4-related genes and pathways identified negative regulation of metabolic process, cellular process, “signal transduction”, “cell growth”, and “epithelial cell proliferation” (as examples), consistent with our theory that decreasing levels of TCF4 lead to less restricted cell growth and more inflammation.

### The biological response of KCs to IL-17C compared with IL-17A.

IL-17C and IL-17A share receptor signaling using the common IL-17RA coreceptor of the IL-17R heterodimeric receptor complex (11), with IL-17C signaling being reliant on IL-17RE and IL-17A reliant on IL-17RC. To better understand the effect of IL-17C in KCs, we generated KOs of each IL-17 receptor subunit (IL17RA, IL17RE, IL17RC KOs) using CRISPR/Cas9 approaches (26) and performed RNA-Seq with and without 8 hours of cytokine stimulation (IL-17C, TNF-α + IL-17C, IL-17A, and TNF-α + IL-17A). Utilizing an FDR of ≤ 0.1 and fold-change of ≥ 2, we determined that IL-17C is less potent at inducing gene expression in KCs compared with IL-17A. Only 87 differentially expressed genes (DEGs) increased following IL-17C stimulation versus 552 by IL-17A stimulation, with 53 genes overlapping. Similarly, 126 DEGs decreased following IL-17C stimulation in contrast to 346 DEGs by IL-17A (fold change > 0; FDR ≤ 0.1) (with 71 genes overlapping) (Figure 3B). Both IL-17A and IL-17C demonstrated significant synergy with TNF-α.

GO term analysis of the DEGs induced by IL-17C identified enriched BPs including “Viral transcription”, “Translational initiation”, “Translation”, “Defense response to fungus”, “Inflammatory response”, “Epidermis development”, and “Keratinization”. IL-17A–induced DEGs showed enrichment for “Keratinization”, Epidermis development”, “Keratinocyte differentiation”, “Inflammatory response”, “Signal transduction”, “Innate immune response”, and “Establishment of skin barrier” (Figure 3C). Analyses of IL-17C and IL-17A in combination with TNF showed similar enriched categories with additional numbers of DEGs within each GO category (Supplemental Figure 7). IL-17C–induced gene expression was largely dependent on IL-17RA and IL-17RE, and IL-17A induced gene expression was dependent on IL17RA and IL-17RC (Supplemental Figure 8), confirming findings by others (reviewed in ref. 4). Comparative examination of our findings support the idea that IL-17C is a less potent proinflammatory mediator in KCs compared with IL-17A but can augment immune responses through synergy with proinflammatory cytokines including TNF-α.

### IL-17C induces ZC3H12A and NFKBIZ expression in an IL-17RA/RE–dependent manner.

**Figure 4:**
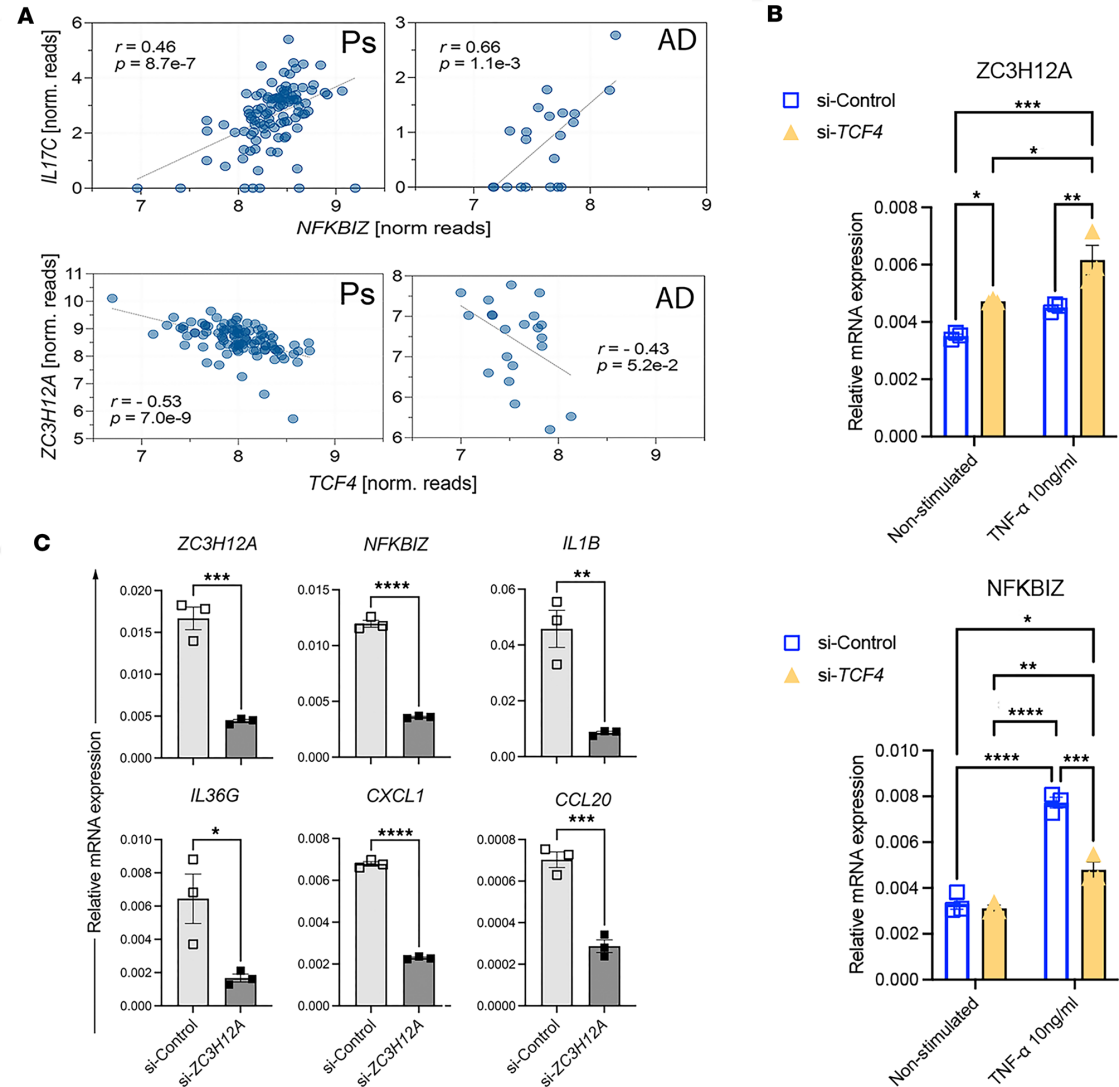
TCF4 negatively regulates ZC3H12A, which promotes keratinocyte inflammation. (**A**) *IL17C* expression positively correlates with *NFKBIZ* expression, and *TCF4* negatively correlates with *ZC3H12A* expression in lesional Ps and AD skin. (**B**) qPCR of *ZC3H12A* and *NFKBIZ* expression in si-*TCF4* keratinocytes reveals a *TCF4*-mediated effect on *ZC3H12A* expression. (**C**) siRNA-mediated silencing of *ZC3H12A* in keratinocytes leads to significant decreases in proinflammatory genes. *n* = 3, mean ± SEM, 2-way ANOVA with post hoc Tukey test (**B**) and 2-tailed Student’s *t* test (**C**). *P < 0.05, **P < 0.01, ***P < 0.001, ****P < 0.0001.

Decreases in TCF4 occur concomitant with increases in IL-17C and ZC3H12A (Figure 2C). RNA-Seq analyses of KCs stimulated with IL-17C identified increases in *NFKBIZ* and *ZC3H12A* that were dependent on IL-17RA/RE (Figure 3, D and E), and both *NFKB1* and *ZC3H12A* were identified in the IL-17C promotor analyses (Figure 1). In Ps and AD lesional skin, *IL17C* positively correlates with *NFKBIZ* (*r* = 0.46, *P* = 8.7 × 10^–7^, Ps; *r* = 0.66, *P* = 0.001, AD; Figure 4A), and *TCF4* negatively correlates with *ZC3H12A* (*r* = –0.53, *P* = 7.0× 10^–9^, Ps; *r* = –0.43, *P* = 0.05, AD; Figure 4A). In each of these diseases, significant increases in IL-17A, IL-17C, and TNF-α occur (27, 28), and these cytokines decrease *TCF4* (Figure 3, A and F). To determine whether *TCF4* regulates *NFKBIZ* or *ZC3H12A*, we silenced *TCF4* in KCs using siRNA and observed increases in *ZC3H12A* but not *NFKBIZ* (Figure 4B). Exposure of *si-TCF4* KCs to TNF-α further increased *ZC3H12A* and led to increases in *NFKBIZ*, further demonstrating the overlapping complexity of the autocrine proinflammatory feedback loop within the KC. Together, these findings support a role for IL-17C signaling and TNF-α signaling in the positive regulation of *ZC3H12A*, the negative regulation of *TCF4*, and additional regulation between *TCF4* and *ZC3H12A*.

Whether ZC3H12A promotes or suppresses inflammation remains unclear (20, 29); thus, we sought to determine the role of ZC3H12A in KCs. Using si-*ZC3H12A* methods, we examined changes in gene expression of several well-known KC inflammatory markers. *ZC3H12A* silencing in KCs results in significant decreases in *ZC3H12A*, *NFKBIZ*, *IL1B*, *IL36G*, *CXCL1*, and *CCL20* (Figure 4C), demonstrating that *ZC3H12A* also promotes inflammation and plays an important role in IL-17C–mediated immune responses. Finally, IL-17C stimulation of KCs also increases *ZC3H12A,* *IL-36G*, *IL-1B*, and *CCL20* (Supplemental Figure 9), further promoting additional autocrine inflammation.

Together, our findings provide evidence that *TCF4* negatively regulates the expression of *ZCH3H12A* and *IL-17C*, and IL-17C decreases *TCF4* and promotes the expression of *ZC3H12A* and *NFKBIZ* in an IL-17RA/RE–dependent manner; this in turn drives the expression of proinflammatory cytokines, chemokines, and signaling responses that work together to create an autocrine proinflammatory feedback loop within the KC.

### TCF4 expression negatively corresponds to skin inflammation in an IL-17C–IL-17RA/RE–dependent manner.

**Figure 5:**
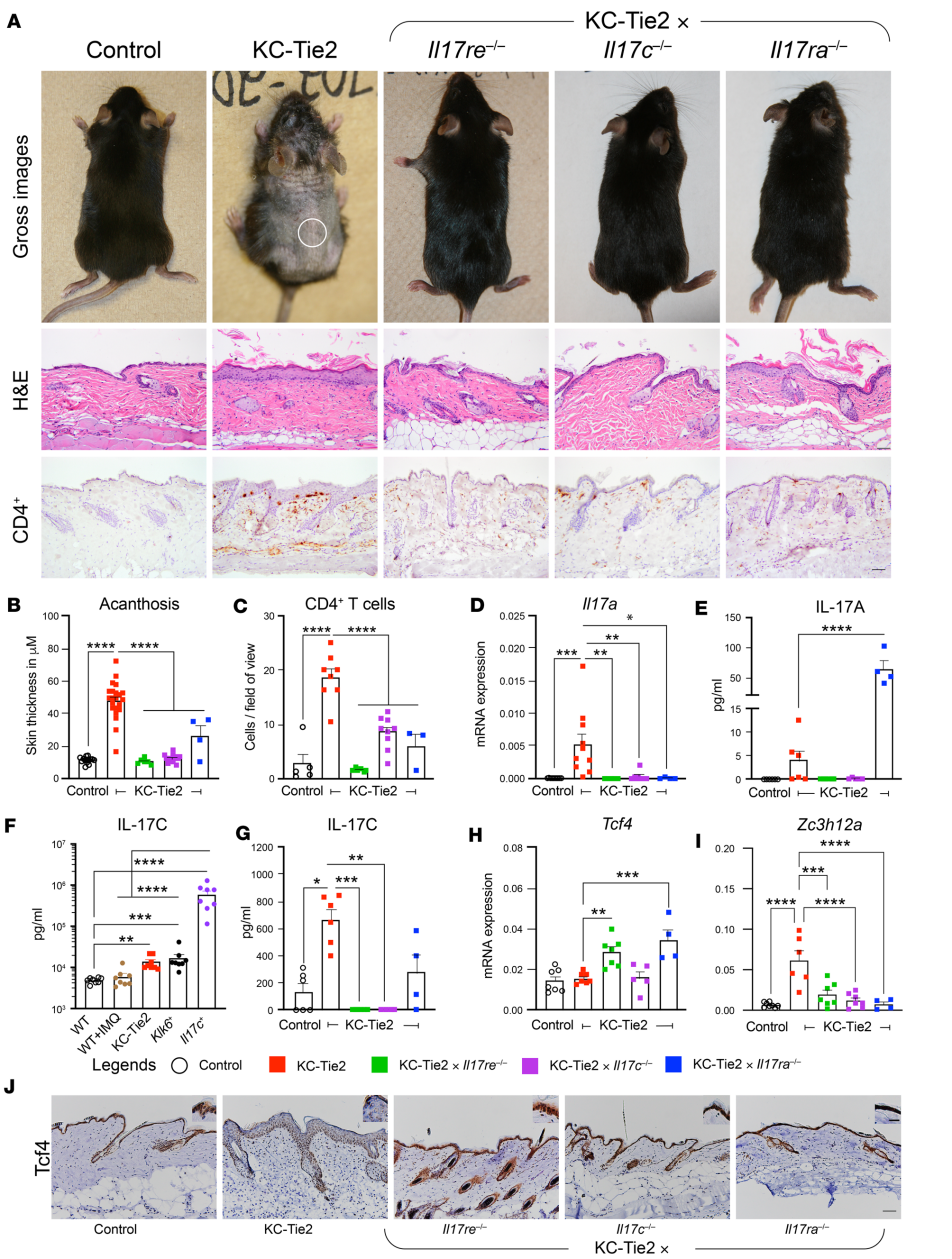
KC-Tie2 mice have significant increases in skin IL-17C. Skin inflammation occurs in an IL-17C–IL-17RA/RE–dependent manner and negatively correlates with Tcf4 expression and positively correlates with *Zc3h12a* expression. (**A**) Images of representative KC-Tie2 mice and KC-Tie2 mice deficient in *Il17c*, *Il17ra*, and *Il17re*. Representative images of H&E-stained skin isolated from the demarcated region of each mouse and adjacent skin stained for CD4^+^ T cells. (**B**) Dot plots of epidermal thickness measures for each mouse (control, *n* = 14; KC-Tie2, *n* = 22; KC-Tie2 × *Il17re^–/–^*, *n* = 6; KC-Tie2 × *Il17c^–/–^*, *n* = 16; KC-Tie2 × *Il17ra^–/–^*, *n* = 4) within each mouse strain. (**C**) Dot plots for individual quantification of CD4^+^ T cell numbers/field of view in skin of each mouse line (*n* = 3–9/group [grp]). (**D** and **E**) qPCR measures for *Il17a* (**D**, *n* = 4–10/grp) and ELISA of IL-17A protein (**E**, *n* = 4–6/grp) expression for individual mice within each group. (**F**) IL-17C protein expression (using ELISA) in skin of psoriasis mouse models (*n* = 8/grp), including imiquimod, KC-Tie2, *Klk6^+^*, and *IL-17C^+^*. (**G**) IL-17C protein expression (using ELISA) for KC-Tie2 mice and KC-Tie2 mice deficient in *Il17c*, *Il17ra*, and *Il17re* (*n* = 4–7/grp). (**H** and **I**) qPCR of *Tcf4* (**H**) and *Zc3h12a* (**I**) gene expression in each mouse line (*n* = 4–7/grp). (**J**) IHC of TCF4 protein in skin shows decreases in nuclear staining in KC-Tie2 mice compared with staining in control (WT) mice and KC-Tie2 mice deficient in *Il17c*, *Il17ra*, and *Il17re*. Insets represents higher-magnification image. One-way ANOVA, followed by post hoc Tukey test; *P < 0.05; **P < 0.002; ***P <0.005, ****P < 0.0001. Scale bar: 50 μm (**A** and **J**) and 25 μm (inset of **J**).

To translate our findings observed in KCs to a whole organism, we took advantage of a mouse model of inflammatory dermatitis, called the KC-Tie2 mouse (Figure 5A). The KC-Tie2 mouse model expresses the tyrosine kinase receptor, Tie2, in keratin 5–positive (K5-positive) KCs. KC-Tie2 mice spontaneously develop an inflammatory skin phenotype that is characterized by acanthosis (thickened epidermis of the skin), an increased presence of T cells, antigen-presenting cells, neutrophils located within the stratum corneum (30), and increases in cutaneous IL-17C, IL-17A, and TNF-α. The phenotype of KC-Tie2 mouse skin improves significantly following systemic interference with drug targets commonly used to treat patients with Ps, including TNF-α (31), IL-23, IL-17A, and IL-17RA (32), and also fails to respond to drugs that patients with Ps do not respond to, including erlotinib and anakinra (33), making it a strong preclinical model of Ps. Confirming prior reports (34), we determined that dorsal skin of adult KC-Tie2 mice develops increases in epidermal thickness (acanthosis; Figure 5B) and increases in the number of infiltrating CD4^+^ T cells concurrent with increases in IL-17A transcript and protein expression (Figure 5, A and C–E) compared with littermate controls (30). Like human Ps lesional skin, nuclear TCF4 staining was decreased in the inflamed skin of KC-Tie2 mice (Figure 5J). Using ELISA, we examined IL-17C protein expression in several Ps animal models of psoriasiform-like dermatitis, including the KC-Tie2 mouse model, the topical imiquimod treated C57BL/6 mice (35), the *Klk6^+^* model (36), and a model in which *Il17c* is genetically overexpressed in KCs (8). Each model had significant increases in cutaneous IL-17C compared with control mice, except the imiquimod model (Figure 5F).

Since the KC-Tie2 mice are well characterized and have significant increases in IL-17C not genetically driven using a keratin promoter, we backcrossed KC-Tie2 mice to *Il17c*-, *Il17re*-, and *Il17ra*-deficient mice. KC-Tie2 mice deficient in either *Il17c*, *Il17ra*, or *Il17re* appeared identical to C57BL/6 animals, had normal epidermal thickness and similar numbers of cutaneous CD4^+^ T cells as control mice, and showed decreases in IL-17C protein back to control mouse levels (Figure 5). *Il17a* mRNA expression returned to control mouse levels in KC-Tie2–*Il17re-*, KC-Tie2–*Il17c-*, and KC-Tie2–*Il17ra-*deficient mice, and IL-17A protein also returned to control mouse levels in KC-Tie2–*Il17re-* and KC-Tie2–*Il17c-*deficient mice but remained increased in KC-Tie2–*Il17ra*-deficient animals (Figure 5, D and E), despite the lack of skin inflammation. This is consistent with prior reports demonstrating that *Il17ra-*deficient mice have elevated IL-17A expression (37, 38), although the elevated IL-17A is unable to exert a biological effect due to the lack of IL-17RA. Others have also shown that dysbiosis in skin of mice lacking IL-17RA in KCs can lead to increases in IL-17A expression (39). *Tcf4* mRNA expression did not decrease in KC-Tie2 mouse skin compared with control mice, nor did it change in KC-Tie2–*Il17c*-deficient mice. However, IHC of TCF4 protein in control versus KC-Tie2 mouse skin revealed decreases in nuclear TCF4 protein expression in KC-Tie2 KCs (Figure 5J), consistent with our observations in Ps lesional skin (Figure 2C). *Tcf4* mRNA levels increased in KC-Tie2–*Il17re-* and KC-Tie2–*Il17ra-*deficient mouse skin (Figure 5H), and IHC of TCF4 confirmed these increases in KCs in KC-Tie2–*Il17re*-, KC-Tie2–*Il17c-*, and KC-Tie2–*Il17ra-*deficient mice to similar levels as control mice (Figure 5J). Finally, *Zc3h12a* expression correlated with measures of skin inflammation, such that *Zc3h12a* increased in KC-Tie2 mice compared with control animals and decreased in the absence of *Il17c*, *Il17re*, and *Il17ra* (Figure 5I). Together these data reveal that cutaneous inflammation in KC-Tie2 mice is regulated by IL-17C–IL-17RE/RA signaling that negatively corresponds with TCF4 expression.

### Tcf4 silencing in Ps mice exacerbates skin inflammation.

**Figure 6:**
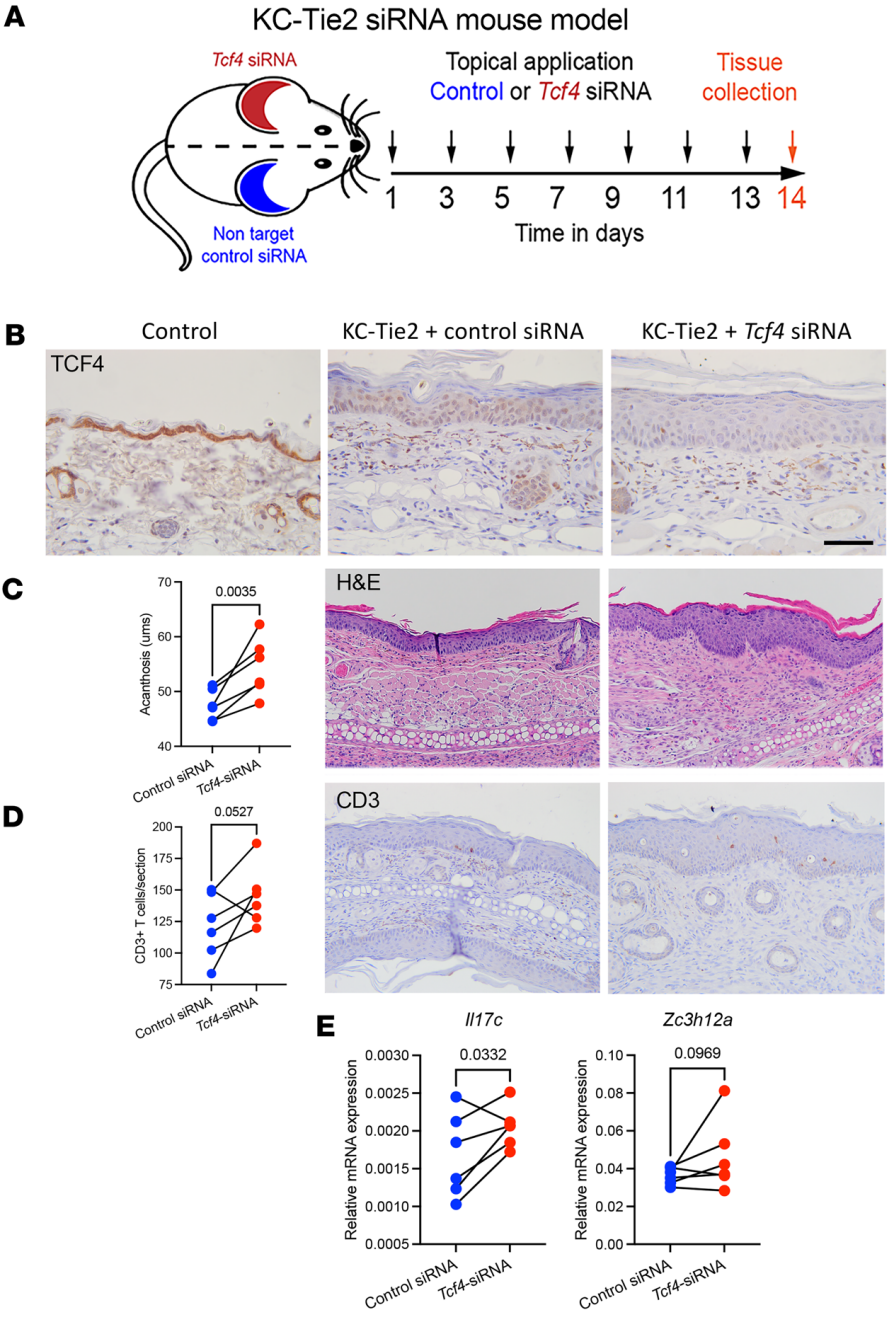
siRNA-mediated *Tcf4* knockdown in KC-Tie2 mouse skin increases *Il17c* and *Zc3h12a* expression and worsens skin inflammation. (**A**) Schematic representation of the approach used to knockdown *Tcf4* expression in KC-Tie2 mouse skin. (**B**) Tcf4 IHC on control and KC-Tie2 mouse skin. Ear skin treated with control siRNA and *Tcf4* siRNA from individual KC-Tie2 mice (*n* = 6) was stained with antibodies against Tcf4. Representative images from paired ear skin shows decreases in Tcf4 nuclear protein expression in KC-Tie2 mice versus control mice and further decreases in Tcf4 in ear skin treated with topical *Tcf4* siRNA compared with ear skin treated with control siRNA. (**C**) Quantification of epidermal thickness measures (ms) and representative images of H&E staining from paired ear skin of KC-Tie2 mice treated with control siRNA or *Tcf4* siRNA. (**D**) Quantification of CD3^+^ T cell counts and representative images of CD3^+^ T cell staining from paired ear skin of KC-Tie2 treated with control siRNA or *Tcf4* siRNA. (**E**) qPCR analyses identifies increases in *Il17c* and *Zc3h12a* expression in paired ear skin treated with *si-Tcf4* compared with si-control. Paired Student’s *t* test. *P < 0.05, **P < 0.005. Scale bar: 25 ms (**B**) and 100 ms (**C** and **D**).

To demonstrate that TCF4 directly affects skin inflammation in vivo, we used a topical delivery method of siRNA to decrease *Tcf4* gene expression (40). Control and *Tcf4* siRNA were applied topically to individual ears of KC-Tie2 mice for a period of 14 days (Figure 6A). Ear skin of KC-Tie2 mice treated with *Tcf4* siRNA developed decreases in epidermal TCF4 expression (Figure 6B) and worsened skin inflammation compared with control siRNA–treated ear skin, including increases in epidermal thickness and CD3^+^ T cell staining (Figure 6, B–D). Quantitative PCR (qPCR) analyses of skin demonstrated significant increases in *Il17c (*1.3-fold, *P <* 0.05) and a trend toward increases in *Zc3h12a* (1.2-fold, *P* > 0.05) expression in ears treated with si-*Tcf4* versus si-control–treated skin (Figure 6E), confirming findings in human KCs (Figure 4B). This demonstrated that *Tcf4* negatively regulated *Il17c* and *Zc3h12a* in vivo to promote skin inflammation.

## Discussion

There are now considerable data contributing to the understanding of the pivotal role of IL-17 cytokines in promoting inflammatory responses, with IL-17A and IL-17F being the most well understood. IL-17C has 23% amino acid sequence homology with IL-17A, and we and others have demonstrated its capacity to promote inflammation (8, 11). However, the mechanisms involved in the regulation of IL-17C and downstream signaling events and how these together promote inflammation remain poorly understood.

IL-17C has been shown to contribute to epithelial immune responses in several inflammatory skin disorders including Ps, AD, and hidradenitis suppurativa (HS; refs. 7, 11, 41). IL-17C is expressed by KCs in the skin in response to pathogen-associated molecular patterns (PAMPs) and proinflammatory cytokines (5, 6, 42). IL-17C can trigger more complex immune responses, as shown in a transgenic mouse model of dermatitis where *Il17c* expression was directed to the epidermis (8). Nonetheless, the mechanisms regulating IL-17C expression and how IL-17C signaling through IL-17RA/RE contribute to inflammatory responses in the skin remain rudimentary. Several studies have demonstrated additive and synergistic effects of IL-17C and other proinflammatory cytokines, including TNF-α, IL-22, and IL-1β in the promotion of inflammation initiated in KCs (5, 6, 8). KC- and immune cell–derived cytokines, including TNF-α, IL-1β, IL-17A, IL-36γ, and IL-17C (8, 10–12), also elicit autocrine production of IL-17C (43). TNF-α was shown to induce IL-17C expression through the p38 mitogen-activated protein kinase (44) and the NF-κB pathway (45). Another study described NF-κB binding sites upstream from the IL-17C promoter (45). We were able to confirm *IL17C* promoter NF-κB binding sites that were enriched in the 1 kb region upstream of genes having *IL17C*-correlated expression in psoriatic skin (FDR = 1.7 × 10^–5^). However, in our study, silencing *NFKB1* did not affect *IL17C* mRNA expression in KCs. Other putative transcription factors including CEBPG and JUN also did not affect *IL17C* expression.

We identified a novel role for the transcription factor TCF4 in the regulation of IL-17C. Our in silico analyses identified TCF4 binding sites in the *IL17C* promoter region (Supplemental Figure 4), corresponding to open chromatin regions in epithelial KCs (Figure 2B). We also determined that TCF4 negatively regulates expression of the RNase ZC3H12A (Regnase-1/MCPIP1), which also increases in response to IL-17C. TCF4 has been shown to have an important role in neurological development and DC diversification in the immune system (46, 47). In the skin, TCF4 appears to have a role in maintaining skin epithelial stem cells through Wnt-dependent and Wnt-independent pathways (17). TCF4-regulated genes identified in a human neuroblastoma cell line associated TCF4 with processes including cell survival, epithelial-to-mesenchymal transition, and neuronal differentiation (48). The finding that TCF4 acts as a negative regulator of IL-17C and ZC3H12A in KCs is potentially novel and may be due to the “opposing” effects of epidermal differentiation and inflammatory responses (49). Our findings that TCF4 negatively regulates inflammation in KCs support findings by others who have shown that loss of *Tcf4* in DCs leads to increases in DC-derived IL-6, IL-23, IL-1β, TNF-β, and IL-12p40, which in turn lead to increases in T cell–derived IL-17A and IFN-γ and exacerbate experimental autoimmune encephalitis (EAE) (50). Similarly, Pozniak and colleagues showed that overexpression of TCF4 leads to decreases in TNF-α and T cell activation and suppression of TCF4 elicits inflammation, including increases in IFNs, thereby increasing the sensitivity of immune checkpoint inhibitors used to treat melanoma (51). Experiments examining direct promotor binding in combination with reporter assays wherein specific target sequences in the IL-17C promotor for TCF4 are mutated will provide important support for these findings. TCF4 expression decreased in lesional skin of patients with Ps and AD, and its protein expression decreased in KC nuclei in Ps skin and in KC-Tie2 mouse skin. *TCF4* also decreased in KCs stimulated with proinflammatory cytokines, including IL-17A, IL-17C, and TNF-α, and decreased in KCs deficient in IL-17RE and IL-17RA. TCF4 decreases negatively correlated with IL-17C and with ZC3H12A, and silencing *TCF4* in human KCs and in KC-Tie2 mouse ear skin led to increases in *IL17C* and *ZC3H12A*, which occurred concomitant with increases in epidermal thickening in ear skin of KC-Tie2 mice.

IL-17C signals through a heterodimeric receptor, composed of IL-17RE and the common IL-17RA chain (11). Prior studies have shown that IL-17C proinflammatory responses overlap with those of IL-17A (5, 8, 43). Our findings provide support for these and further characterize this overlap, as well as identify unique IL-17C– and IL-17A–induced genes in a more comprehensive manner and clearly demonstrate that IL-17C evokes a less robust response compared with IL-17A. Similar to IL-17A, IL-17C augments gene expression in synergy with TNF-α, suggesting that both IL-17A–and IL-17C–TNF-α synergism is critical for skin immune response. Indeed, the findings of autocrine KC inflammation derived directly from decreases in TCF4, and paracrine inflammation elicited by immune-cell derived cytokines like IL-17A-TNF-α, and subsequent TCF4 decreases argue for a complex regulatory relationship that occurs within and between KCs and immune cells within an inflamed environment. The elimination of the skin phenotype in KC-Tie2 animals following deletion of *Il17c*, *Il17re*, and *Il17ra* confirms the criticality of IL-17C–IL-17RE/RA signaling in mouse dermatitis and validates the capacity of using biologics that target the IL-17 common receptor, IL-17RA, as an approach that encompasses inhibiting not only IL-17A/F but also IL-17C. The increases in TCF4 in these mice further validate the capacity for skin inflammation to regulate TCF4 expression, with KC data suggesting that TNF-α, IL-17A, and IL-17C likely contribute to regulating (decreasing) TCF4. The elimination of IL-17C on its own also eliminated skin inflammation supporting prior work showing that antibody treatment with anti–IL-17C improves inflammation in acute models of Ps and AD (9). However, the therapeutic potential of blocking IL-17C for inflammatory skin diseases has not been fully explored. One small trial (*n* = 25 patients) examined clinical improvement in patients with AD following treatment with anti–IL-17C antibody (MOR106) (clinicaltrial.gov, NCT02739009). Although early-stage results from this trial suggest the possibility of efficacy, these studies ultimately failed to meet expected primary outcome measures and have not been pursued. Additional clinical trials examining IL-17C inhibition in skin disease have not been pursued, although reports of efficacy of anti–IL-17RA treatment following failed anti–IL-17A treatment are thought to result from interfering with IL-17C–IL-17RA signaling (52). Whether directly targeting IL-17C inhibition to treat Ps would be effective remains unclear.

Lastly, we found *NFKBIZ* and *ZC3H12A* to be the most significant genes induced by IL-17C in human KCs, with their induction being dependent on IL-17RE and IL-17RA. *NFKBIZ* is an established Ps susceptibility locus (53) and has been shown to play a critical role in IL-17F and IL-17A proinflammatory responses (54, 55). The *ZC3H12A* gene has also been linked to Ps by genetic association studies (56) and encodes the MCP-1–induced protein 1 (MCPIP1, also called Regnase-1), which has been shown to play a vital function in regulating inflammatory responses. IL-17A has been shown to induce ZC3H12A/MCPIP1 expression in KCs and is highly dependent on the STAT3 pathway (57). ZC3H12A belongs to a family that also includes Tristetraprolin (TTP) and roquin, which regulate RNA splicing, polyadenylation, export, translation, and decay (58–60) and serves as a negative regulator of inflammatory responses (61–63). ZC3H12A/Regnase-1 has been previously shown to suppress imiquimod-elicited skin inflammation by regulating IL-17A and IL-17C responses (20). Thus, ZC3H12A/Regnase-1 suppresses inflammation and provides negative feedback to regulate inflammatory responses. Our findings, in contrast, indicate that *ZC3H12A* promotes inflammation and are consistent with those of others (29), who recently demonstrated that IL-17A induces Regnase-1 phosphorylation, leading to reduced degradation of Regnase-1–targeted mRNA production, and that sustained phosphorylation (beyond that required for degradation) promotes the expression of IL-17A target genes. These findings were dependent on IL-17R signaling (Act1-TBK1/IKK). Thus, it is likely that similar events occur within our system, thereby promoting IL-17C–IL-17RA/RE–ZC3H12A–dependent inflammation within KCs.

In conclusion, our data demonstrate that *TCF4* negatively regulates *IL-17C* and *ZC3H12A* expression in human KCs and in a mouse model of dermatitis, and that IL-17C promotes the expression of *ZC3H12A* and *NFKBIZ* in an IL-17RA/RE–dependent manner and together work collectively to promote inflammation that becomes self-sustaining. These findings identify a role for TCF4 in the negative regulation of IL-17C and ZC3H12A, which promotes an inflammatory feedback cycle within the KC involving NFKBIZ that becomes self-sustaining. These data provide additional information on the biology of IL-17C and provide resources that will help future work on this unique epidermal proinflammatory cytokine.

## Methods

### Sex as a biological variable.

Males and females were used in the human and animal experiments. N/TERT KCs were derived from male skin.

### IL17C promoter analyses.

Promoter analyses were performed using the UCSC GRCh38/hg38 genome version and were based upon the longest annotated IL17C transcript (NM_013278) with transcription start site (TSS) on the (+) strand of chromosome 16 at coordinate 88638571. We performed analyses using TSS-proximal regions 1–10 kb upstream from the TSS (hg38 coordinates, 88628571–88639571). The set of 191 IL-17C–correlated genes was identified by evaluating expression across 99 lesional skin samples from patients with Ps RNA-Seq samples described by us previously (19) (GEO accession nos. GSE54456 and GSE63979). These 99 samples had been sequenced with 80 bp reads using the Illumina Genome Analyzer. Mapped sequence reads were tabulated to obtain counts for each human protein-coding gene as described previously (64). Mapped counts were converted to log_2_-based expression intensities using the voom transformation (65). Semiparametric generalized additive logistic models (GAM) were used to identify DNA motifs enriched in regions upstream of the 191 IL-17C–correlated genes (66). The screen included 2,935 binding sites known to interact with transcription factors or unconventional DNA binding proteins (67). Similar analyses were performed for TCF4.

### N/TERT cell culture and RNA interference.

N/TERTs, an immortalized primary epidermal KC cell line (68), were grown in KC-SFM medium (17005-042, Thermo Fisher Scientific) supplemented with 30 μg/mL bovine pituitary extract, 0.2 ng/mL epidermal growth factor, and 0.3 mM calcium chloride. N/TERTs are referred to as KCs throughout the paper. Targeting siRNA and control siRNA (D-001910-01-20) were purchased from Dharmacon company and introduced into cells by Delivery medium according to the manufacturer’s instructions. Targeting siRNA used in this study were: *TCF4* (Accell human *TCF4* siRNA, E-004594-00-0005), *NFKB1* (Accell human *NFKB1* siRNA, E-003520-00-0005), *CEBPG* (Accell human *CEBPG* siRNA, E-011608-00-0010); *JUN* (Accell human JUN siRNA, E-003268-00-0005), and *ZC3H12A* (Accell human ZC3H12A siRNA, E-014576-00-0010).

### Generation of CRISPR-KO lines in N/TERT KCs and cytokine stimulation.

Guide RNAs were developed using a web interface for CRISPR design (https://www.zlab.bio/resources). The pSpCas9 (BB)−2A-GFP (PX458) was a gift from Feng Zhang (Broad Institute, Cambridge, Massachusetts, USA; Addgene plasmid no. 48138) and was used as a cloning backbone. Using an established CRISPR/Cas9 protocol (26) *IL17RA-*, *IL17RC*-, and *IL17RE*-deficient KCs were generated. Confluent control and IL-17R–deficient KCs were treated with recombinant human IL-17A (20 ng/mL, R&D Systems) and IL-17C (200ng/mL, R&D Systems) with and without TNF-α (10 ng/mL, R&D Systems) for 8 hours prior to RNA extraction.

### Mouse lines and siRNA.

KC-Tie2 mice on a C57BL/6 background was generated as previously described (34). *Il17c-*, *Il17re-*, and *Il17ra-*deficient mice (all C57BL/6) were provided by Amgen and were individually mated with K5tTA and TetosTie2 mice, which were then mated together following the same protocol used to generate KC-Tie2 mice (30). Male and female adult mice were used for all studies. ON-TARGETplus siRNA SMARTpool for mouse *Tcf4* (Accell mouse Tcf4, gene ID: 21413) and nontargeting pool (control siRNA) were purchased from Dharmacon. A preparation for topical application was made that included 2.5 nM siRNA/2 μL of Lipofectamine 3000 (Invitrogen)/10 mg of over-the-counter generic hand cream. Topical siRNA targeting Tcf4 or control siRNA was applied to ear skin of KC-Tie2 mice (Figure 6A) every other day for 14 days. This approach enables control siRNA and Tcf4 siRNA to be applied onto the skin of the same mouse, allowing for a paired statistical analysis and eliminating potential confounding factors that could elicit changes, including microenvironment, cage, and littermate variability. Mice were euthanized the day after the last application, and skin was collected for histology and RNA analyses.

### RNA extraction, qPCR, and RNA-Seq.

Total RNA was isolated using RNeasy plus kit (74136, Qiagen), and qPCR was performed on a 7900HT Fast Real-time PCR system (Applied Biosystems) with TaqMan Universal PCR Master Mix (4304437, Thermo Fisher Scientific). Human primers (Thermo Fisher Scientific) used in this study were: TCF4, Hs00162613_m1; IL-17C, Hs00171163_m1; JUN, Hs99999141_s1;CEBPG, Hs01922818_s1; IL1B, Hs01555410_m1; IL36G, Hs00219742_m1; CCL20, Hs00355476_m1; CXCL1, Hs00236937_m1; S100A7, Hs00161488_m1; DEFB4, Hs00175474_m1; IVL, Hs00846307_s1; FLG, Hs00856927_g1; LOR, Hs01894962_s1; SPRR2A, Hs03046643_s1; ZC3H12A, Hs00962356_m1; NFKBIZ, Hs00230071_m1; and RPLP0, Hs99999902_m1. Mouse primers (Thermo Fisher Scientific) used in this study were: Tcf4, Mm00443210_m1; Il17a, Mm00439618_m1; Il17c, Mm00521397_m1, Zc3h12a, Mm00462535_g1; Nfkbiz, Mm00600522_m1; Zc3h12a, Mm00462535_g1; and GAPDH, Mm99999915_g1. Gene expression was determined by normalizing the gene of interest to that of the housekeeping gene as follows: 2^CT^ (housekeeping gene)/2^CT^ (gene of interest). All human samples were normalized to *RPLPO*, and all mouse samples were normalized to *GAPDH*. Individual housekeeping genes were chosen by comparing differences in CT values between experimental groups for 18S, actin, GAPDH, and RPLPO. The gene that changed the least between groups was chosen.

Libraries for RNA-Seq were generated from polyadenylated RNA and sequenced at 6 libraries per lane on the Illumina Genome Analyzer IIx. TopHat2 was used to align RNA-Seq reads to the human genome, using annotations of GENCODE as a gene model. HTSeq was used to quantify gene expression levels; normalization and differential expression analyses were performed by DESeq2. GO analysis of RNA-Seq data was performed using DAVID (The Database for Annotation, Visualization, and Integrated Discovery, available at https://david.ncifcrf.gov/).

### IHC and ELISA.

Formalin-fixed, paraffin-embedded tissue slides obtained from patients with Ps and healthy controls were heated at 60°C for 30 minutes, deparaffinized, rehydrated, and epitope retrieved with tris-EDTA (pH 9). Slides were treated with 3% H_2_O_2_ (5 minutes), blocked with 10% goat serum (30 minutes), and incubated with primary antibodies against TCF4 (HPA025958-100UL, Sigma-Aldrich), IL-17C (AF1234, R&D Systems), and rabbit IgG isotype control (NI01, Sigma-Aldrich) overnight at 4°C. Slides were then washed and treated with appropriate secondary antibodies, peroxidase (30 minutes), and diaminobenzidine substrate. Slides were visualized with Zeiss Axioskop 2 microscope. Mouse skin tissues were prepared and mounted onto slides as previously described (30) and were stained with H&E or were stained using antibodies targeting CD4 (550280, BD Pharmingen) and TCF4 (22337-1-AP Proteintech). All acanthosis measures and T cell counts were collected by an individual blind to mouse genotype. ELISA for mouse IL-17A (M1700, R&D Systems) and IL-17C (OKBB00438, Aviva Systems Biology) was performed according to individual manufacturer instructions. Skin protein was isolated using RIPA buffer (89900, Thermo Fisher Scientific) with protease and phosphatase inhibitors (PPI) (78442, Thermo Fisher Scientific) at a 1:100 (PPI/RIPA) dilution.

### Single cell ATAC-Seq from human skin.

Skin biopsies (4 mm) were taken and incubated in 0.4% dispase overnight to separate the epidermis and dermis. After the separation, the epidermis was transferred to 0.25% Trypsin-EDTA + 10 unit/mL DNase mixture and incubated at 37°C for 1 hour. The epidermis mixture was then quenched with FBS and precipitated by centrifugation. Cell pellets were then resuspended in PBS + 0.04% BSA. Cell numbers were counted at this step for future dilution calculation. The nuclei isolation protocol was carried out as described by 10X Genomics. The cell lysis efficacy was determined by Countess II FL Automated Cell Counter. The single-cell ATAC-Seq library was prepared by Advanced Genomics Core at the University of Michigan. In total, 10,000 nuclei/sample and 25,000 reads/nuclei were targeted, and the libraries were sequenced using NovaSeq SP 100 cycle flow cell. The raw data were first processed by the Chromium Single cell ATAC Software Suite (10X Genomics) and analyzed using the Signac package in R. Briefly, the single-cell ATAC-Seq data go through a series of analyses including quality control, dimension reduction, clustering, and integration with previously annotated single-cell RNA-Seq data. DNA accessibility profile was then visualized in different cell types and samples.

### Statistics.

Calculations were made using GraphPad Prism Version 9.2 (GraphPad Software). A unpaired 2-tailed Student’s *t* test was used to compare 2 groups, and either a 2-way or 1-way ANOVA with Tukey or Dunnett’s post hoc tests were used for multiple comparisons (as indicated in each figure legend). For paired comparison of ear skin outcomes in mice treated with siRNA, a paired, 1-tailed Student’s *t* test was used. *P* ≤ 0.05 was considered statistically significant. For RNA-Seq data, FDR ≤ 0.1 was used to control for multiple testing.

### Study approval.

Human samples were obtained from volunteer patients with inflammatory skin disease and healthy controls with informed written consent before inclusion in the study in accordance with Declaration of Helsinki principles. All protocols were approved by the University of Michigan IRB. All animal experiments were approved by the Case Western Reserve University IACUC and conformed to the American Association for Accreditation of Laboratory Animal Care guidelines.

### Data availability.

The RNA-Seq data are available at the GEO repository via the GEO accession no. GSE260642. Values for all data points in graphs are reported in the Supporting Data Values file.

## Author contributions

YJ, WRS, RSG, LCT, ACB, EM, JMK, JEG, and NLW designed the research studies. YJ, DG, CZ, CG, CS, RSG, JW, SLS, MKS, RU, AK, XX, OP, EX, and ACB conducted experiments and acquired data. YJ, WRS, RSG, LCT, SLS, EM, JEG, and NLW analyzed data. WRS, WB, RSG, and NLW made the figures. NLW and JEG wrote the manuscript.

## Supplementary Material

Supplemental data

Supporting data values

## Version 1 03/12/2024

In-Press Preview

## Version 2 04/22/2024

Electronic publication
