# Supplementary material for: Suppression of TCF4 promotes a ZC3H12A-mediated self-sustaining inflammatory feedback cycle involving IL-17RA/IL-17RE epidermal signaling
Source: JCI Insight. 2024 Mar 12;9(8):e172764. doi: 10.1172/jci.insight.172764 (PMC11141873; doi:10.1172/jci.insight.172764)
Supplement: Supplemental data [file jciinsight-9-172764-s033.pdf]

Suppression of TCF4 promotes a ZC3H12A-mediated self-sustaining inflammatory feedback cycle involving IL-17RA/IL-17RE epidermal signaling

Yanyun Jiang[1,2], Dennis Gruszka[3], Chang Zeng[1], William R. Swindell[4], Christa Gaskill[5], Christian Sorensen[5], Whitney Brown[5], Roopesh Singh Gangwar[3], Lam C. Tsoi[1], Joshua Webster [3], Sigrun Laufey Sigurdarsottir[3], Mrinal K. Sarkar[1], Ranjitha Uppala[1], Austin Kidder[1], Xianying Xing[1], Olesya Plazyo[1], Enze Xing[1], Allison C. Billi[1], Emanual Maverakis[6], J. Michelle Kahlenberg[7], Johann E. Gudjonsson[*1], Nicole L. Ward [*3,5,8]

1. Department of Dermatology, University of Michigan, Ann Arbor, 48109, MI, USA
2. Department of Dermatology, Sun Yat-sen Memorial Hospital, Sun Yat-sen University, Guangzhou 510120, China
3. Departments of Nutrition and Dermatology, Case Western Reserve University, Cleveland, OH, 44106, USA
4. Department of Internal Medicine, University of Texas Southwestern Medical Center, Dallas, TX, United States.
5. Department of Dermatology, Vanderbilt University Medical Center, Nashville, TN 37232 USA
6. Department of Dermatology, University of California Davis School of Medicine, Sacramento, California, 95616, USA.
7. Division of Rheumatology, Department of Internal Medicine, University of Michigan, Ann Arbor, MI, 48109, USA
8. Vanderbilt Institute for Infection, Immunology, and Inflammation (VI4) and Vanderbilt Center for Immunobiology (VCI), Vanderbilt University Medical Center, Nashville, TN

* Corresponding authors: Nicole L. Ward, PhD, email: nicole.ward@vumc.org or Johann E. Gudjonsson MD, PhD, e-mail: johanng@med.umich.edu

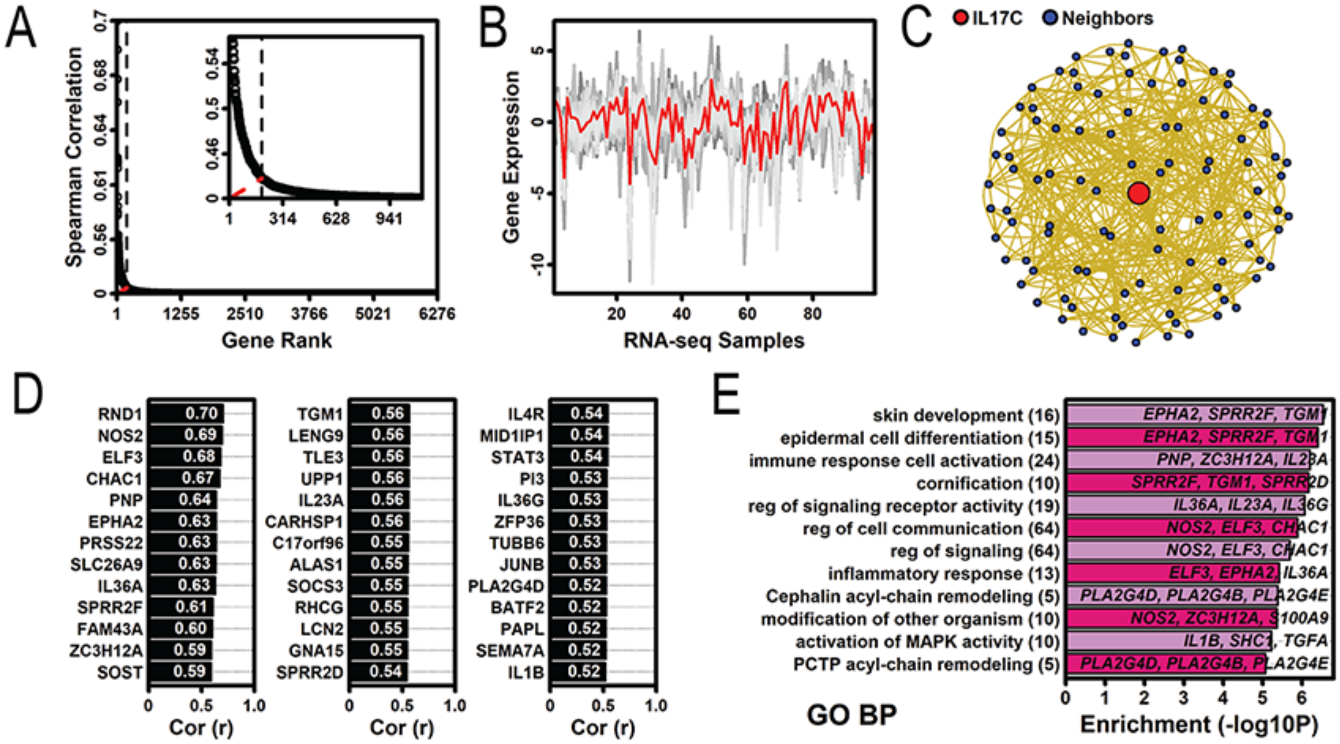

Supplemental Figure 1. *IL17C* correlated gene analysis. (A). Network thresholding analysis identified 6276 genes positively correlated with *IL17C* expression and with detectable expression in at least 5% of PP skin samples (n = 99). The ranked list of genes is plotted on the X axis against the correlation coefficients on the Y axis. The red line represents the minimal distance between the lower-left origin and correlation curve, which defines a set of 191 genes having *IL17C*-correlated expression (rs ≥ 0.41). (B). Normalized expression of the above 191 IL17C-correlated genes (Y axis; red line represents IL17C expression) is shown in the 99 PP skin samples (X axis). (C). Network plot of *IL17C*-correlated genes with *IL17C* at center. (D). Network genes strongly correlated with IL17C are shown with their respective correlation coefficient (r). Gene Ontology Biological Process (GO BP) enriched with respect to the 191 *IL17C*-correlated genes. The number of genes associated with each GO-term are shown (parentheses, left margin) and exemplar genes are listed within the figure.

A

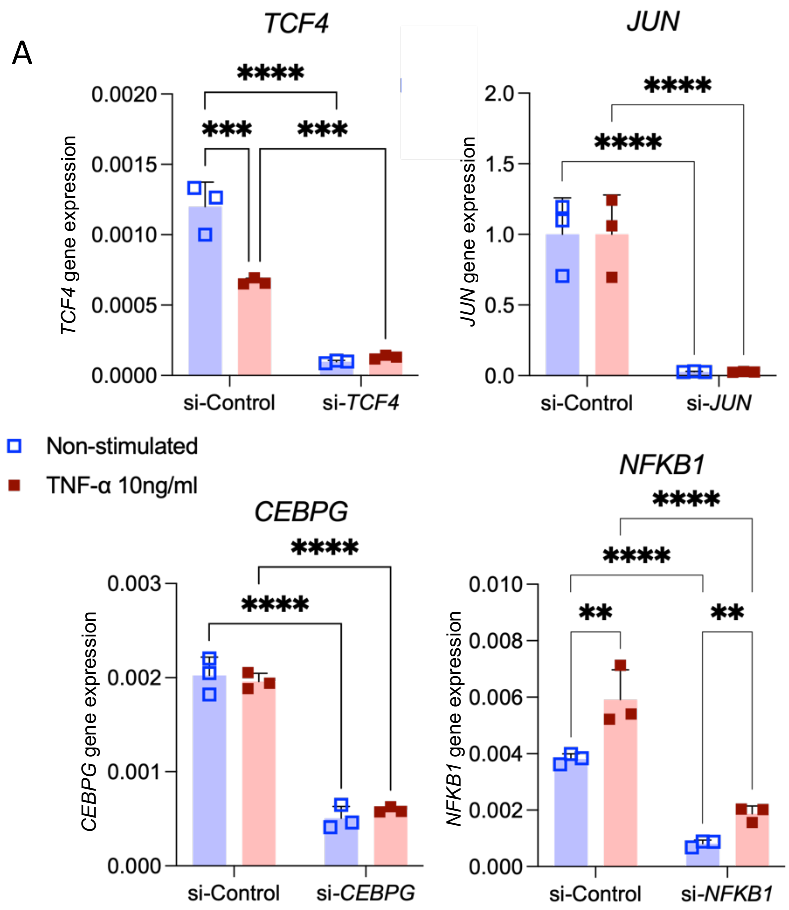

B

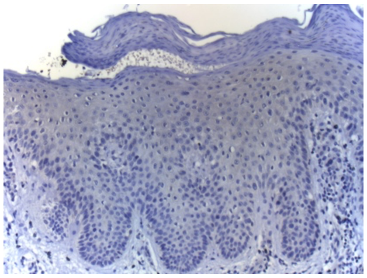

Supplemental Figure 2. Confirmation of siRNA knockdown and Isotype staining control. (A) Quantitative RT-PCR confirms decreases in *TCF4*, *NFKB1*, *JUN* and *CEBPG* gene expression following siRNA-gene specific mediated knockdown in human keratinocytes (N/TERT, n=3, mean ± SD). TNF-α stimulation (10ng/ml for 8hrs) decreases *TCF4* expression and increases *NFKB1*. Two-way ANOVA with post-hoc Tukey test, *$p < 0.05$; ** $p < 0.01$, *** $p < 0.005$ **** $p < 0.0001$. (B) Control rabbit isotype staining on lesional psoriasis skin.

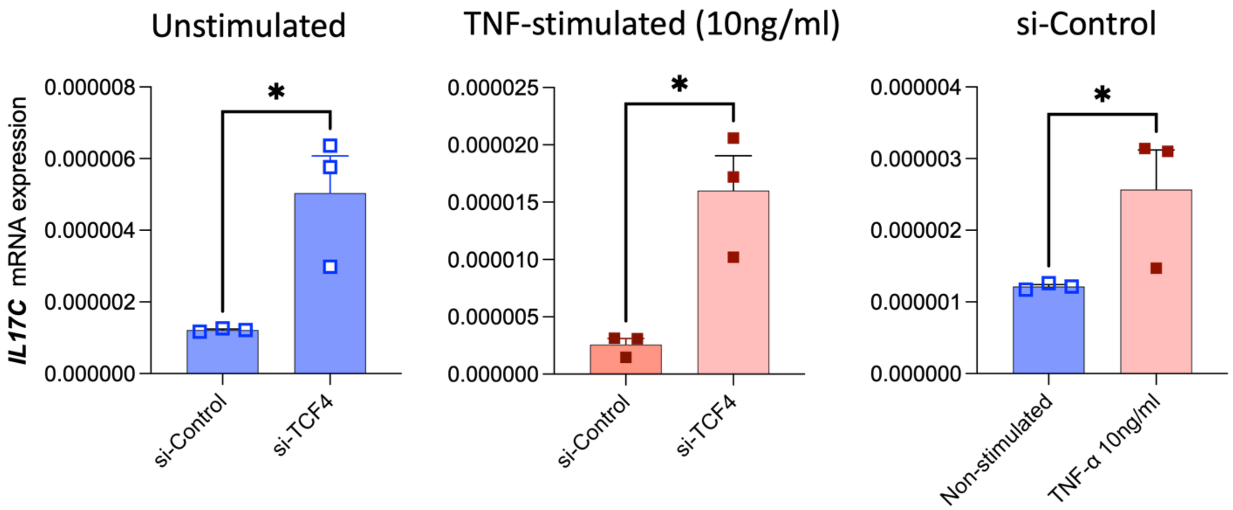

Supplemental Figure 3. Additional statistical comparison of groups presented in Figure 2A. Quantitative RT-PCR of *IL17C* following siRNA-gene specific mediated knockdown in keratinocytes (N/TERT, n=3, mean ± SEM) with and without TNF-α stimulation (10ng/ml for 8hrs). Unpaired T-test, $*p < 0.05$.

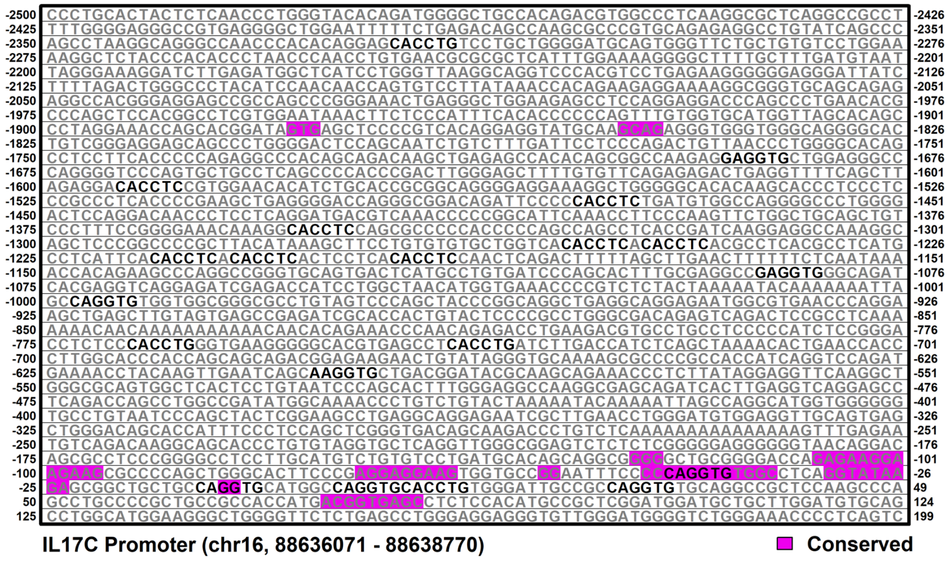

Supplemental Figure 4. IL17C promoter TCF4 binding sites. A binding site (5-CAGGTG/CACCTG-3) known to interact with TCF4 was significantly enriched in 2 kb regions upstream of genes having IL17C-correlated expression in PP skin (P = 5.40E-09, FDR = 1.47E-06). The IL17C promoter sequence is shown with TCF4 binding sites in bold-faced font. Conserved sequences (PhastCons ≥ 0.20) are highlighted (magenta color).

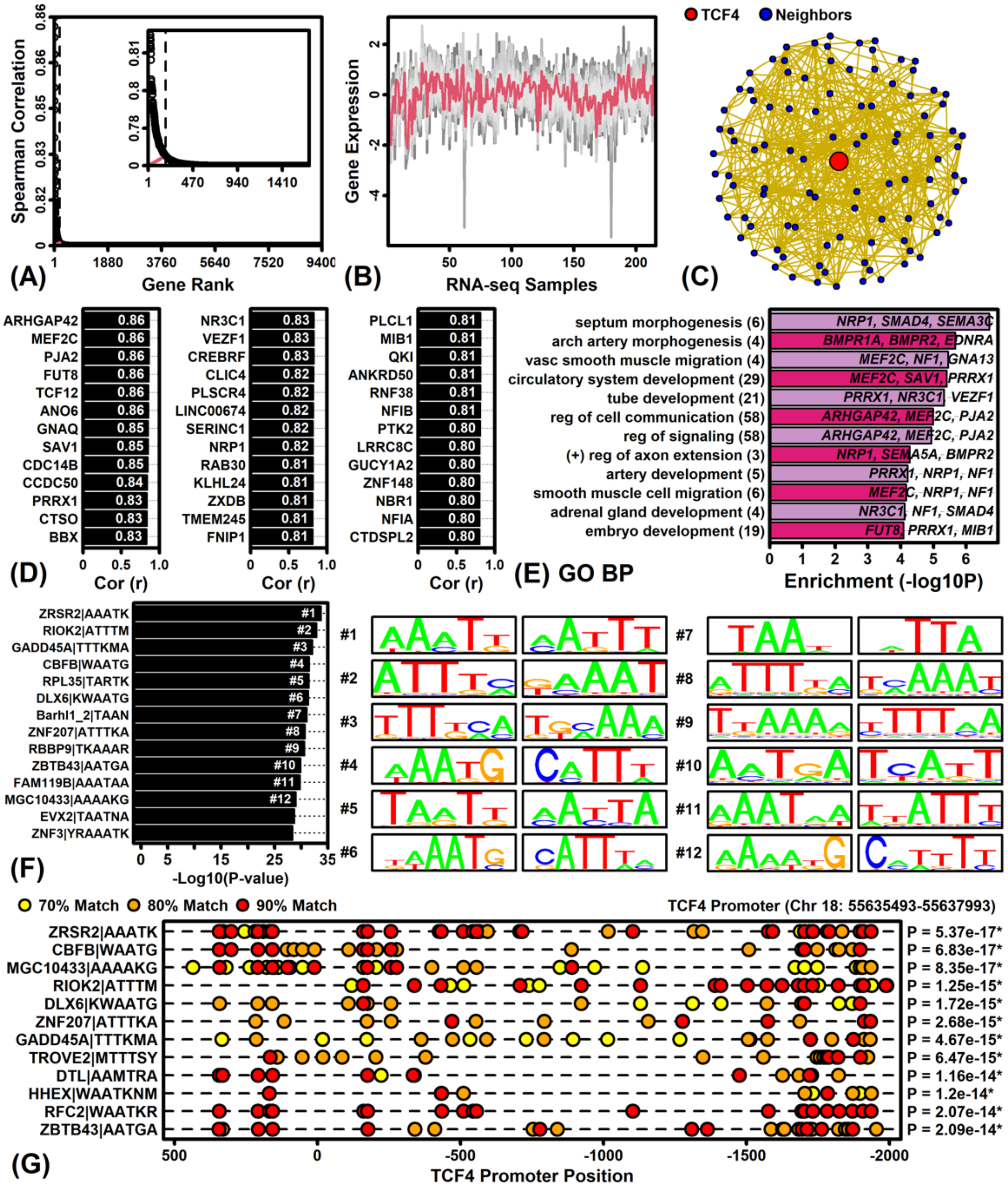

Supplemental Figure 5. ***TCF4*-coexpressed genes and upstream motifs.** (A) Network thresholding. The analysis identified 9400 genes positively correlated with *TCF4* expression and with detectable expression in at least 5% of PP skin samples ($n = 99$). The figure shows correlation coefficients (vertical axis) relative to the ranked list of 9400 genes (horizontal axis). The vertical axis is exponentially scaled (r^{28}) to emphasize higher correlations. The red line represents the minimal distance between the lower-left origin and correlation curve, which defines a set of 184 genes having *TCF4*-correlated expression ($r_s \geq 0.74$). (B) *TCF4*-correlated gene expression. Normalized expression (vertical axis) is shown across the 99 PP skin samples (horizontal axis) for the 184 *TCF4*-correlated genes (red line: *TCF4* expression). (C) *TCF4*

network. *TCF4*-correlated genes are plotted in a spherical network with *TCF4* at center. The top 3 intra-network correlations were used for each gene to draw connections. (D) Top *TCF4*-correlated genes. Network genes most strongly correlated with *TCF4* are shown. (E) GO BP terms. The chart shows GO BP terms most strongly enriched with respect to the 184 *TCF4*-correlated genes. The number of genes associated with each term are shown (parentheses, left margin) and exemplar genes are listed within the figure. (F) DNA motifs. The chart (left) shows motifs most heavily enriched in 5 kb regions upstream of *TCF4*-correlated genes. Only motifs with at least 5 sites in the *TCF4* 5 kb upstream region are shown. Sequence logos for the 12 most significant motifs are shown (right). (G) *TCF4* promoter sites. Top-ranked motif matches are shown within the *TCF4* promoter sequence (interval: -2 kb, 0.5 kb). Matches are shown at three levels of stringency (70% = weakest match; 90% = strongest match).

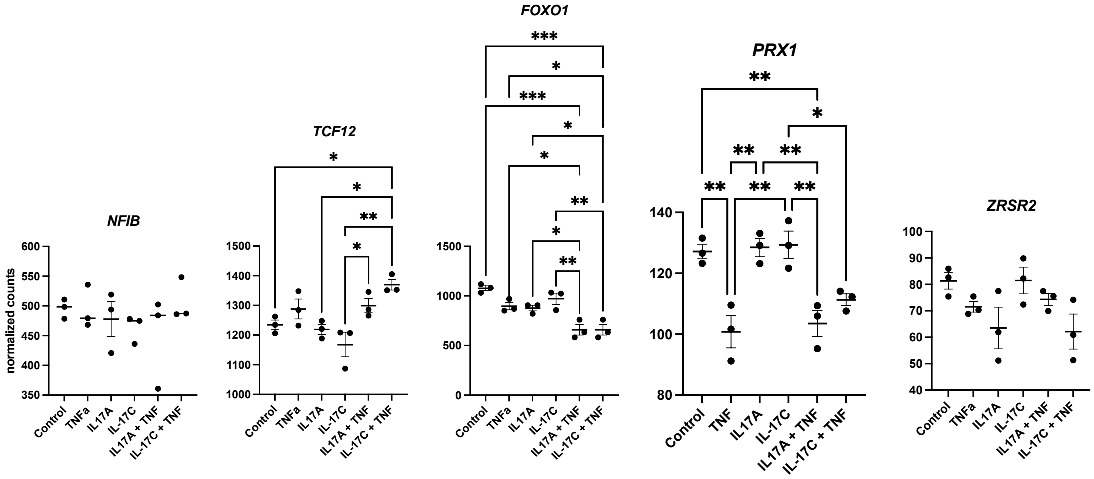

Supplemental Figure 6. Gene expression changes (normalized counts) in predicted TCF4 transcription factors following stimulation of keratinocytes (N/TERT) IL-17C (200ng/ml) and IL-17A (20ng/ml) in keratinocytes with or without TNF-α stimulation (10ng/ml; 8hrs). n=3, mean ± SEM, one-way ANOVA with post-hoc Tukey test, * p <0.05, ** p < 0.002, *** p < 0.0005.

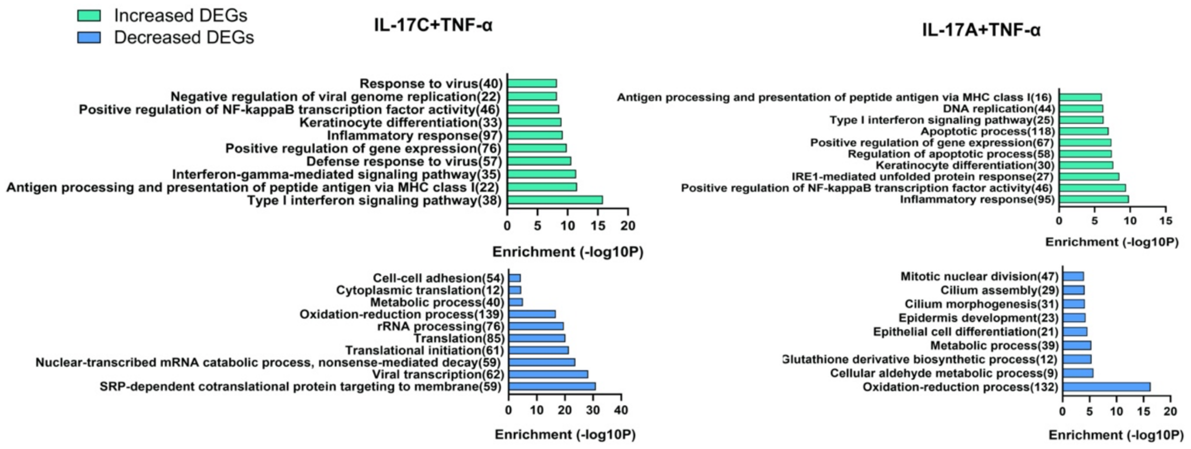

Supplemental Figure 7. GO BP chart shows functional categories enriched in N/TERTs induced by IL-17C and IL-17A when combined with TNF-α stimulation.

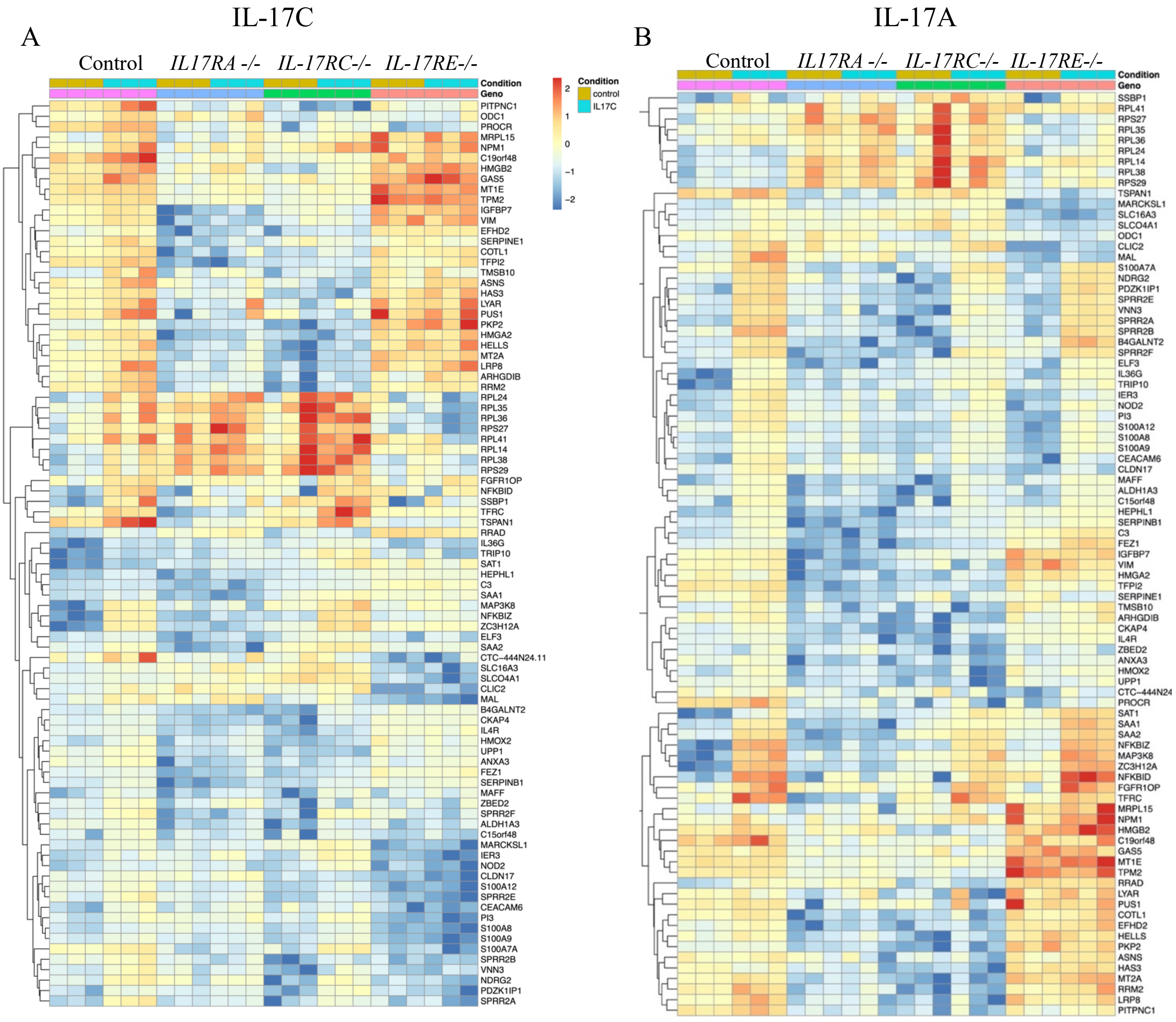

Supplemental Figure 8. Heatmap shows expression level of IL-17C (A) and IL-17A (B) related genes in IL-17 receptor knockout N/TERTs.

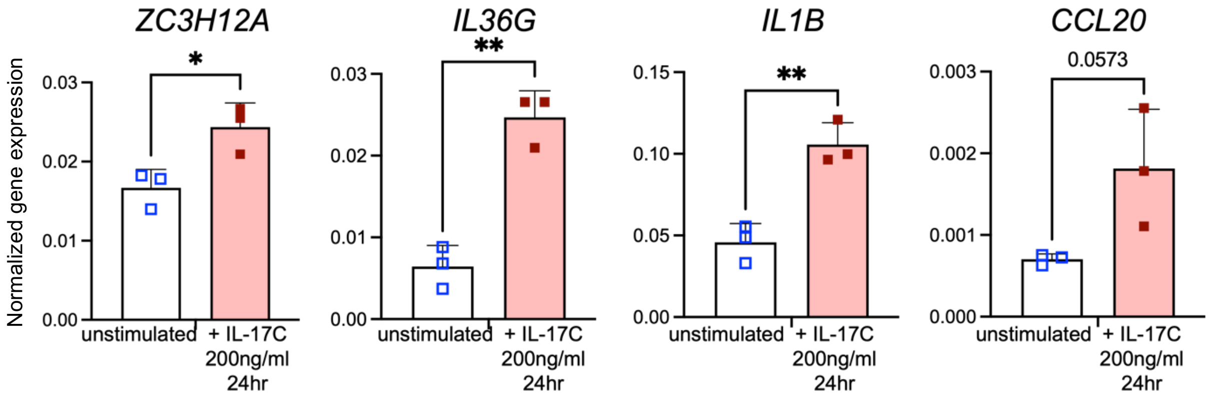

Supplemental Figure 9. Gene expression changes in response to IL-17C stimulation. Quantitative RT-PCR identifies increases in *ZC3H12A*, *IL36G*, *IL1B* and *CCL20* in NTERTs following stimulation with IL-17C (200ng/ml, 8hrs, n=3, mean ± SEM). Unpaired student T-test, *$p < 0.05$; ** $p < 0.01$.
